# Field Deployable Method for Gold Detection Using Gold Pre-Concentration on Functionalized Surfaces

**DOI:** 10.3390/s20020492

**Published:** 2020-01-15

**Authors:** Agnieszka Zuber, Akash Bachhuka, Steven Tassios, Caroline Tiddy, Krasimir Vasilev, Heike Ebendorff-Heidepriem

**Affiliations:** 1 Institute for Photonics and Advanced Sensing, School of Physical Sciences, The University of Adelaide, Adelaide 5005, Australia; agnes.zuber@gmail.com; 2 Deep Exploration Technologies Cooperative Research Centre, School of Physical Sciences, The University of Adelaide, Adelaide 5005, Australia; steven.tassios@csiro.au (S.T.); caroline.tiddy@unisa.edu.au (C.T.); 3 ARC Center of Excellence for Nanoscale BioPhotonics, The University of Adelaide, Adelaide 5005, Australia; 4 CSIRO, Process Science and Engineering, Gate 1, Normanby Road, Clayton 3169, Australia; 5 Future Industries Institute, University of South Australia, Mawson Lakes 5095, Australia; krasimir.vasilev@unisa.edu.au; 6 School of Engineering, University of South Australia, Mawson Lakes 5095, Australia

**Keywords:** surface chemistry, plasma polymerization, salinization, gold sensing

## Abstract

Gold in a rock is usually associated with other elements, forms nuggets, or is hosted within the crystal lattice of a mineral (e.g., pyrite) and is often heterogeneously distributed and trapped inside the rock matrix even after crushing. Gold can be liberated from these rock matrices by chemical leaching, but then their concentration becomes too low for detection by a portable method due to the dilution effect of the leaching process. In this paper, we present a proof-of-concept method for gold pre-concentration to enable the detection of gold in rock at low levels using a portable technique. Two coating methods, plasma polymerization (PP) and wet chemistry (WC), were utilized to generate surface coatings, which were then compared for their effectiveness in binding gold ions. Laser-induced breakdown spectroscopy (LIBS) was used as a portable technique for the detection of immobilized gold on these modified surfaces. The detection limit for pure gold ions in solution incubated on PP and WC coatings was determined to be as low as 80 ppb. To demonstrate the real-life capability of the method, it was tested for rock sample leachates bearing 300–500 ppb gold.

## 1. Introduction

Gold has been a major target for profitable geological exploration for centuries. It is usually heterogeneously distributed and trapped inside rock particles. Due to its low natural abundance and inhomogeneous distribution, gold has to be detected at a very low concentration and in large volume. Gold in a rock can be detected either with liberation of the gold from the rock particles it is trapped in, which can be achieved using chemical methods, or without liberation using a physical method with high penetration depth. One of the oldest and most commonly used methods employed for the liberation of gold is a fire assay which uses different chemical methods for sample preparation and pre-concentration and then utilizes a physical technique for actual gold measurement such as inductively coupled plasma mass spectrometry (ICP-MS) [1]. Although, the fire assay technique allows for the detection of gold at low ppb levels in rocks, it is time-consuming and requires multistep processing which involves high temperature and hazardous reagents disabling the portability of the method. A physical technique which does not require liberation of gold due to its high penetration depth is the gamma activation technique [2,3,4], which involves high-energy X-rays to excite the atomic nuclei of the gold atom into gold isomer. Subsequently, the gamma radiations emitted from the resting nuclei are detected for gold detection. This technique provides rapid, precise and low level (ppm) measurement of gold in rocks, but it involves high energy particles generated from both X-ray and gamma rays which hinders the portability of the technique on site.

To address these issues, a lot of research has been focused on developing new portable physical techniques. Current portable techniques include X-ray fluorescence (XRF) and laser-induced breakdown spectroscopy (LIBS), which are capable of detecting gold [5,6]. However, these techniques have small penetration depth into the sample, which makes it hard to measure sample with heterogeneous gold distribution and deeply trapped gold. Therefore, there is growing interest to develop a new portable method to overcome these limitations. We hypothesize that the pre-concentration of gold in solution by binding it onto a functionalized substrate facilitates low-level gold sensing using currently available portable physical techniques. It has been well known that gold (III) chloride [7] and gold thiosulfate [8] ions as wells as gold nanoparticles (NPs) [9,10,11,12] bind to a surface functionalized with amines groups. 

Traditional methods for the surface modification of substrates with amine groups utilize wet chemistry (WC) approaches. These approaches use classical reactions and simple laboratory equipment with most of the analytes being in a liquid phase. In the past few decades, plasma polymerization (PP) has been demonstrated as a powerful technique for surface functionalization [9,10,12,13,14,15]. The fabrication of functional PP films for immobilization of gold/silver NPs and heavy metal ions have been employed for different applications such as antibacterial surfaces [16], modulation of cellular responses [17] and removal of heavy metal ions [18]. PP utilizes a collection of ions, excited atoms, molecules, free radicals, protons, and neutral species from a wide range of precursors to form polymer-like coatings on a surface. The plasma technique enables the fast deposition of a polymer (few minutes) with no production of chemical waste. Key benefits of the technique include its applicability to any type of substrate material and no requirement for surface pre-modification [11,19]. As a comparison, most WC techniques (e.g., self-assembled monolayers) require specific substrate material [20]. The PP process is also solvent free, which is beneficial in terms of cost, since it does not produce waste solvents [12]. 

In this paper, we report the proof-of-concept of a field-deployable method for low-level gold sensing by pre-concentrating the gold in solution onto a planar substrate, and then employing physical technique of LIBS for gold detection. LIBS was chosen due to its portability and use of light compared to the use of high energy radiation/particles for XRF and gamma activation method. Furthermore, LIBS can only be used to measure small sample volume which makes it challenging to measure samples with heterogeneous element distribution [21,22]. To overcome this obstacle, we report a chemical method for leaching that does not use toxic and hazardous chemicals, opening up the possibility for the field-deployability of all processes, including chemical leaching of rock, gold pre-concentration onto functionalized substrates, and finally detection with portable LIBS. Overall, the proposed method is easy to handle, requires small amount of sample and is easily transportable, which makes it better than the commercially available techniques such as fire assays and gamma radiation assay. Our method’s capability for gold detection was demonstrated with pure gold solutions containing gold ions or gold NPs, while real world applicability was assessed with rock samples.

## 2. Results and Discussions

### 2.1. Coating Thickness

Both surface functionalization techniques resulted in thin, nanoscale film coatings. (3-Aminopropyl)triethoxysilane (APTES) is hydrolytically unstable in air, leading to the formation of multilayer polymer whose thickness increases with time due to uncontrolled hydrolysis [15]. Therefore, WC coatings in this study were prepared in a controlled, anhydrous environment, resulting in a coating thickness of 6 nm. In comparison, WC coatings prepared in ambient air using the same incubation time were reported to have twice larger thickness [15]. In contrast to WC, PP produces much thicker coatings, with allylamine-based plasma polymer growing tens of nm per minute at 10 W [23]. The thickness of the PP coatings in this study increased with polymerization time; 60, 180, and 300 s polymerization time resulted in 17, 30 and 50 nm coating thickness, respectively. A 30-nm PP coating thickness was selected for sensitivity investigation as similar coating thickness was previously found to successfully bind differently sized gold NPs [10,24,25]. 

### 2.2. Chemical Analysis of the Coatings via XPS

The C1s, N1s, O1s, and Si2p bands of the survey spectra were analyzed to determine the elemental composition of the coatings. The area of each band normalized to the sum of the areas of these four bands was used as a measure of the concentration of the element corresponding to the band. 

**Figure 1:**
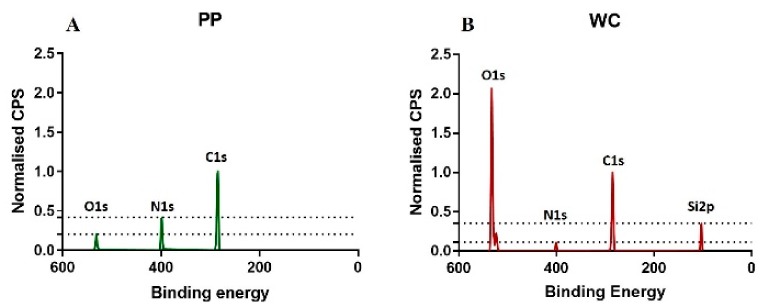
XPS survey spectrum of PP (**A**) and WC (**B**) coatings on glass substrate samples shown as counts per second (CPS) vs bind-ing energy. The background was subtracted, and each graph was normalized to C1s.

The carbon C1s, nitrogen N1s and oxygen O1s bands were found in the spectra of all functionalized surfaces, but silicon Si2p band was only found for the WC coating (Figure 1). APTES monomer used for the WC coating contains 9C, 1N, 3O and 1Si in one molecule, which gives a percent ratio of C:N:O:Si of 64:7:22:7. However, for the WC coating, a percent ratio of 48:3:32:17 was measured. This discrepancy between monomer and polymer coating shows that the portion of silicon relative to carbon is lower for the APTES monomer molecule but higher for the polymer coating, indicating that the Si band in the XPS spectrum is not only originating from the APTES-based coating but also from the glass used as a substrate. This result is supported by the penetration depth (10 nm) of XPS, which is deeper than the WC coating thickness (6 nm).

The percent ratio of the elements C, N, O and Si of the PP coating is 78:17:26:0. The allylamine monomer used for PP coatings does not contain silicon. The absence of silicon in the XPS spectrum agrees with the XPS penetration depth (10 nm) being smaller than the PP coating thickness (30 nm). As the allylamine monomer does not contain oxygen, the oxygen found for the PP coating indicates oxidation, which was also observed for the WC coating. 

The larger thickness of the PP coating relative to the WC coating correlates with the higher carbon content of the PP coating relative to the WC coating. The higher nitrogen concentration for the PP coating compared to the WC coating correlates with the higher portion of nitrogen in an allylamine molecule compared to an APTES molecule. 

Deconvolution of C1s carbon band was used to determine the content of the functional groups on the surface. According to Bachhuka et al. [26], the C1s band consists of four peaks with the following binding energies:

285.0 eV: aliphatic carbon (C-H or C-C)
286.1 eV: amines (primary C-NH_2_, secondary (C-NH-C), traces of C-O bonds due to oxidation
286.6 eV: imines (C=N), traces of nitrile (C≡N)
288.2 eV: C=O bonds due to contamination

**Figure 2:**
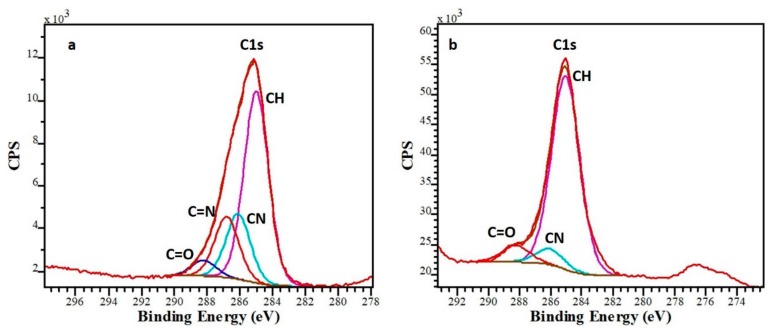
C1s band deconvolution of the PP (**a**) and WC (**b**) coatings.

The C1s band was fitted with these four peaks by fixing the position of each peak at the binding energies given above and making all peaks to have the same band width (Figure 2). The percentage of each peak was determined via normalization to the total area of the C1s band. The normalized areas of the peaks at 286.1 eV and 286.6 eV were considered as a measure for the amount of amine and imine groups in the coating, i.e., the number of groups that can bind gold. As the PP coating contains five times more amine and imine groups than the WC coating (Table 1 and Figure 2a), it is anticipated to be able to bind more gold. 

### 2.3. Zeta Potential and PH of the Gold Solutions

The high negative zeta potential of the citrate capped gold NPs in solution (−34.6 ± 2.02 mV) indicates a strong negative charge for the NPs, resulting in a good colloidal stability of the NPs due to higher repulsion than attraction forces between the NPs [27]. The value of the zeta potential for the 50 nm citrate capped NPs of this work is close to the value reported for 17.3 nm citrate capped NPs (−39.5 mV) [28].

The pH of the gold NP solution is neutral, while the pH of the solutions containing gold ions (dissolved AuCl_3_ or gold nugget and rock leachates) is much lower (Supplementary Table S3). The amines on the surface of the coatings become protonated at low pH, with the amount of the protonation increasing with decreasing pH [29,30], enabling enhanced electrostatic interaction between negatively charged gold species (ions and citrate capped NPs) and positively charged surface. 

### 2.4. Detection of Gold for Pure Gold Samples

**Figure 3:**
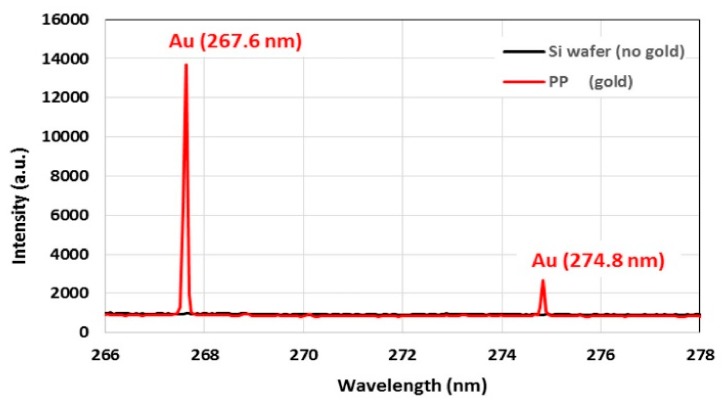
The LIBS intensity of PP coated substrate incubated with gold ions (red) and uncoated substrate without gold used as control sample (black).

The binding ability of gold ions and NPs to the coatings was determined by measuring the amount of gold bound to the coatings using LIBS. The strongest gold emission peak is at 267.6 nm, shown for a PP coated wafer substrate incubated with gold ions (red) compared to the control coated wafer, which was not incubated with gold (black) in Figure 3. Gold ion and NP solutions with different gold concentrations were used to determine the limit of detection (LOD), which is the minimum quantity of a signal that can be reliably detected. LOD with respect to LIBS gold peak area was calculated as the sum of the mean and three times standard deviation of the control samples, which were PP and WC coated substrates with no gold incubated on them. The LIBS signal related LOD was measured to be 9.18 gold peak area. The gold concentration related LOD is defined as the concentration of gold in the solution, for which the LIBS gold signal of the substrate incubated with this solution is above the LOD value of LIBS, i.e., the minimum gold concentration in solution with a LIBS gold peak area >9.18.

**Figure 4:**
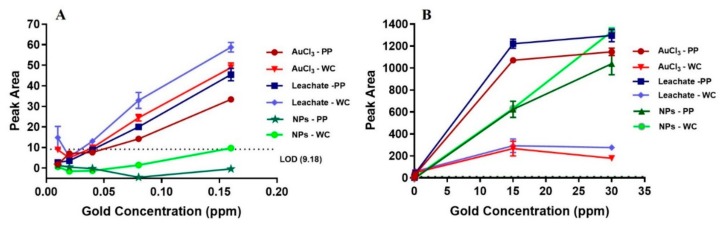
LIBS signal intensity of PP and WC coatings incubated with gold ion and gold NP solutions as a function of low (**A**) and high (**B**) gold concentration of the solutions. LOD is shown as dotted line.

Figure 4 shows LIBS signal intensity as a function of low and high gold concentration for gold ion solutions (AuCl_3_ and gold nugget leachate) and gold NP solution for PP and WC coatings. For solutions containing low concentration of gold (10–160 ppb), the LIBS signal of gold ions is much higher than for gold NPs. More precisely, gold NPs cause only non-zero LIBS signal for 160 ppb gold solution incubated on WC coating, whereas gold ions show linear increase of LIBS signal for >10 ppb gold solutions incubated on both PP and WC coatings. The LOD of gold ions in solution incubated on PP and WC coatings is 80 ppb, whereas the LOD of NPs is much higher: 1 ppm for PP coating and 15 ppm for WC coating. For solutions containing high concentration of gold ions (15–30 ppm), the LIBS signal of gold ions on the PP coating is 4–6 times higher compared to the WC coating and reaches saturation at 30 ppm gold in solution. By contrast, a high concentration of gold NPs (15–30 ppm) causes a similar linear increase of LIBS signal with no indication of saturation for both PP and WC coatings.

These LIBS results of gold ions and NPs can be explained by the following factors: volume of gold per surface coverage, availability of binding sites on the surface and protonation of amine groups. The volume of gold per surface coverage and availability of binding sites (i.e., the availability of free surface without gold) were estimated using the simple model that each ion or NP bound to the surface occupies a square that is determined by the ion or NP diameter, D, i.e., each ion or NP covers an area of D^2^ on the substrate surface (4 cm^2^). These ions and NPs are assumed to occupy a regular square grid (Supplementary Figure S1). All ions and NPs in the solution volume used for incubation (1 mL) are assumed to be bound to the surface, i.e., to contribute to the surface coverage, provided there is sufficient surface area available.

The surface area covered by gold ions and NPs is calculated from the number of gold species in the incubation volume and the area a single species covers. The surface coverage, C, is defined as the covered surface normalized to the maximum available surface area of the substrate (4 cm^2^). Details of the calculations are given in the supplement model 1. Table 2 shows the surface coverage for different concentrations of the gold ion and NP solutions used for incubation.

### 2.5. Binding of Gold Ions on PP and WC Coatings

The model shows that, for gold ions, excess free surface (i.e., C<<1) is available for low concentrations <1 ppm, predicting that the availability of binding sites (amine and imine groups) does not play a role in this concentration rage. This prediction agrees with PP and WC coatings incubated with <1 ppm gold ion solution showing similar linear increase of the LIBS signal intensity with gold concentration and the same LOD, despite the PP coating having more amine and imine groups. For high gold ion concentrations of 15–30 ppm, the incubation volume contains more gold ions than can be bound to the surface via a monolayer on a square grid (i.e., C >1), predicting the binding of gold ions on the surface (i.e., LIBS signal intensity) saturates and is determined by the availability of binding sites. This is in agreement with saturation observed at 30 ppm gold ion concentration for both PP and WC coatings. The higher LIBS signal for the PP coating is consistent with the higher amount of amine and imine groups (acting as binding sites) for the PP coating compared to the WC coating. Specifically, the four-fold higher N/C ratio for the PP coating correlates with the 4–6 times higher LIBS signal for the PP coating relative to the WC coating at high concentrations of 15 and 30 ppm gold ion solutions.

### 2.6. Binding of Gold on PP And WC Coatings

In contrast to gold ions, large excess of free surface (i.e., C<<1) is available for NPs, even at concentration of 15–30 ppm, predicting that availability of binding sites does not play a role for NPs and therefore similar LIBS signal intensity is found for gold NPs on both PP and WC coatings for low and high gold NP concentration, which is indeed observed. Furthermore, the gold NPs do not show any sign of saturation in agreement with the large amount of available binding sites.

### 2.7. Comparison of Binding of Gold Ions and NPs

Gold ions and NPs show different binding behavior; at low concentrations gold NPs show lower LIBS signal and at high concentration gold NPs shows no saturation but linear increase compared to gold ions. The different behavior of the gold NPs relative to gold ions is attributed to two effects that decrease rate of binding; protonation and large size of gold NPs:

i The higher pH of the gold NP solution relative to the gold ion solutions results in a lower protonation of amine groups, which hinders binding compared to the high protonation effect of low-pH gold ion solutions.
ii The size of gold species has been demonstrated to affect the rate of their binding on a coating [25,31,32]. The larger gold NPs bind slower than the smaller gold ions, leading to smaller number of bound NPs compared to ions at the same incubation time.

The reduction in binding rate has to be substantial to explain the low LIBS signal for gold NPs at low concentration for the following reason. Our model predicts that the volume of gold per surface coverage scales with the diameter of the species on the surface (0.32 nm for gold ions and 50 nm for gold NPs). Therefore, the gold volume per surface coverage is two orders of magnitude larger (157 times) for NPs than that for gold ions, making the binding of gold NPs more than 2–3 orders of magnitude slower compared to gold ions if protonation effect on binding rate is negligible. The slower binding rate of gold NPs also agrees with absence of saturation for NPs at 30 ppm compared to ions. Assuming gold NPs can reach the same surface coverage than gold ions, saturation of NPs is expected at 157 times higher LIBS signal compared to gold ions due to larger volume of gold NPs at the same surface coverage.

### 2.8. Leaching of Rock Samples

The effectiveness of gold binding for real application was determined using rock standards from Rocklabs, such as SP72, SN74, HiSilP3, SQ88, OREAS991, and OREAS62e. These samples were chosen due to their difference in gold content and mineralogy (Supplementary Info S1). The efficiency of HClO to leach gold and other elements was calculated as the ratio of measured element concentration in the rock leachates relative to the expected element concentration in the rock leachates assuming complete leaching (based on Rocklabs and OREAS data). The gold leaching efficiency for the rocks varied between 47% and 92% (Table 3). The leaching efficiency of other elements was studied for OREAS62e and OREAS991 rock leachates. Both leachates contained partially leached aluminium (9–17% leaching efficiency) and magnesium (31%–76% leaching efficiency). Iron and sulphur leached completely in all analyzed rocks.

### 2.9. Libs Measurment of PP and WC Coatings Incubated with Rock Leachate

**Figure 5:**
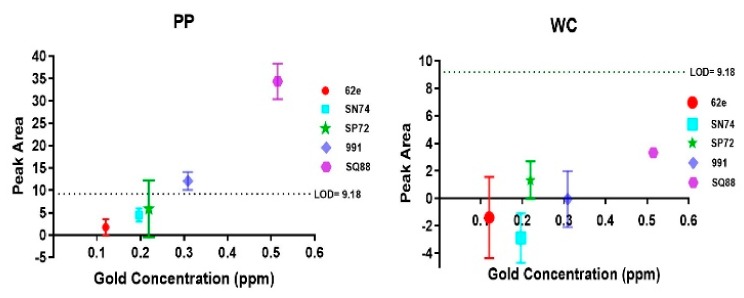
LIBS gold peak area for rock leachates incubated on PP and WC coatings.

For WC coatings, no gold above LOD was detected for any of the rock samples investigated. For PP coatings, gold above LOD was detected for rock samples with highest gold concentration in the leachate (Figure 5): OREAS991 rock leachate (309 ppb gold) and SQ88 rock leachate (515 ppb gold). These results indicate that the pre-concentration of gold on PP coating combined with LIBS allows for the detection of >300 ppb of gold in rock leachate.

The LIBS signal of coatings incubated with rock leachates is attenuated significantly in comparison to the LIBS signal of coatings incubated with pure gold leachate or AuCl3 solution. This is attributed to the binding of other elements found in the leachates (Supplementary Table S1). For example, both Al and Mg were detected by LIBS on PP and WC coatings incubated with OREAS62e and OREAS991 leachates. High concentrations of iron were found on all WC coatings incubated with rock leachates, particularly for rock samples SN74, OREAS62e, SP72. Some iron was also detected on the PP coating incubated with SP72 rock leachate.

Firstly, as the number of functional groups is limited, binding of additional elements reduces the number of available binding sites for gold ions, which hampers the binding of gold ions. Secondly, the additional elements on the coatings attenuate the LIBS signal, reducing the signal to noise ratio. Both the reduced binding efficiency and the lower signal-to-noise ratio for the same gold concentration make the gold LOD for rock leachates (~300 ppb) three times higher than that of the pure gold leachate (80 ppb). 

**Figure 6:**
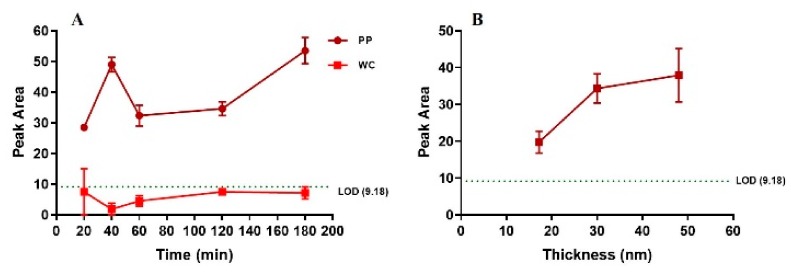
LIBS gold peak area of coatings incubated with SQ88 rock leachate as a function of incubation time for 30 nm PP coating and 6 nm WC coating (**A**) and as a function of coating thickness for PP coatings at 60 min incubation time (**B**).

The detection of gold as a function of incubation time of rock leachate on PP and WC coatings was investigated for the SQ88 rock leachate (515 ppb gold) (Figure 6A). For the WC coating, long incubation time did not result in detection of gold above LOD. For the PP coating, binding of gold was observed after 20 min of incubation, but did not change significantly with longer incubation time, indicating that 20 min is sufficient to bind gold above LOD in a rock leachate on PP coating. 

The binding efficiency of the SQ88 rock leachate was also tested for PP coatings with different thicknesses (17, 30, and 50 nm). The detected gold increased from 17 nm to 30 nm thickness, and then levelled for 50 nm thickness. This suggests the minimum coating thickness to achieve saturated LIBS gold signal is 30 nm for PP coating (Figure 6B).

### 2.10. Measurment of Rock Powder Samples by Using Libs

To validate the necessity of our proposed gold detection method, i.e., leaching and pre-concentration of gold onto functionalized surfaces, the direct LIBS measurement of rock powder samples without leaching was performed. Two types of rock powder samples were prepared for LIBS measurement: a) rock powders directly deposited on adhesive tape and b) rock powder samples prepared by suspending 1g of rock powder in 1mL of water and incubating these suspensions on PP and WC coated surfaces. No gold was detected for the rock powders on adhesive tape (Supplementary Figure S2) or for the WC and PP coated surfaces incubated with rock powder suspensions (Supplementary Figure S3 and S4) even for the sample bearing 47 ppm of gold (OREAS991). This is attributed to the low chance of targeting the laser against the rock particles bearing gold, which are also likely to be surrounded by particles containing elements of higher abundance. The absence of the LIBS gold signal for the two rock powder samples highlights the significance of leaching and pre-concentration of gold on a functionalized surface, to achieve the detection of gold for low gold concentration samples with a portable LIBS instrument. 

## 3. Materials and Methods

### 3.1. Substrates

Two types of substrates were used for surface functionalization: glass cover slip for X-ray photoelectron spectroscopy (XPS) and silicon wafer for ellipsometry and LIBS. As silicon wafers exposed to air exhibit a thin amorphous silica layer on their surface, the surface chemistry of glass and silicon wafer are considered to be comparable. As silica wafers are non-transparent (unlike glass coverslip), they are particularly suited for ellipsometry [33]. All surfaces were sonicated in acetone and ethanol for 30 min each before coating. 

### 3.2. Plasma Polymerisation (PP)

Allylamine was used as monomer for PP due to its known stability in water, which is essential for practical applications. The PP was carried out in an in-house built reactor consisting of glass-walled cylindrical chamber with internal electrodes and a 13.56 MHz plasma generator. The substrates were cleaned for 2 min at 50 W with air plasma. PP coatings were deposited on the substrates using 10 W power, 0.11 mbar pressure and 60, 180, or 300 s plasma run time. 

### 3.3. Wet Chemistry (WC)

For WC based silanization, widely used monomer (3-Aminopropyl) triethoxysilane (APTES) [14,15,16] was employed. The WC coatings were prepared in an anhydrous environment. Clean substrates were immersed in a solution of 50 mM APTES in anhydrous toluene and then incubated for 1 hour at room temperature in a nitrogen-purged glovebox. Afterwards, the WC coated samples were washed twice with anhydrous toluene, moved to the fume hood, and sonicated in anhydrous toluene for 10 min. Finally, the WC coated samples were dried with nitrogen and heated at 90 °C for 2 h. 

### 3.4. Gold and Rock Samples

To determine the effectiveness of binding gold to the functionalized surfaces, three different types of pure gold samples were investigated: 50 nm citrate capped gold NPs from Nanocomposix suspended in MiliQ water, gold ions in HClO leachate of gold nuggets and gold ions in AuCl_3_ aqueous solution (AuCl_3_ powder purchased from Sigma dissolved in MiliQ water).

The effectiveness of gold binding for real application was determined using rock standards from Rocklabs, such as SP72, SN74, HiSilP3, SQ88, OREAS991, and OREAS62e. These samples were chosen due to their difference in gold content and mineralogy (Supplementary Table S1). 

### 3.5. Liberation and Pre-Concentration of Gold Species

Gold and rock samples were leached in 4.8% HClO, which was prepared by adding sodium hypochlorite into 10% NaCl and decreasing pH down to 5 with glacial acetic acid. 5.11 mg of gold nugget was leached in 5 mL of HClO within 30 min. 1 g of rock powder was leached in 35–38 mL of HClO (except 110 mL of HClO used for rock OREAS991) for 1 h under continuous agitation with a magnetic stirrer followed by 2 mL 32% HCl (10 mL for OREAS991) for 15 min. The rock leachate samples were filtered using a Minisart cellulose acetate 0.45 µm syringe filters. For pre-concentration, 1 mL of leached gold solution was placed on a PP or WC coated substrate with area of 21 mm × 21 mm, leaving a dry margin of about 1 mm width to prevent the solution from flowing off the surface. The solutions were incubated for 1 hour and then washed in MilliQ water and dried with nitrogen. 

### 3.6. X-RAY Photoelectron Spectroscopy (XPS)

XPS was used to determine the concentration of elements and functional groups on PP and WC coated surfaces not incubated with any solution. All spectra were recorded using a Spec SAGE XPS spectrometer (SPECS Surface Nano Analysis GmbH, Berlin, Germany) equipped with a monochromatic Mg radiation source operated at 10 kV and 20 mA. Atomic compositions of the samples were identified from survey spectra recorded over a 0–1000 eV with pass energy of 100 eV at a resolution of 0.5 eV. All binding energies were corrected relative to C1s carbon peak at 285.0 eV. Processing and curve fitting were performed using CasaXPS.

### 3.7. Ellipsometry

A variable angle spectroscopic ellipsometer (J. A. Woollam Co. Inc., Lincoln, NE, USA) was used to determine the thickness of the PP and WC coatings on silicon wafers. Calibration was performed using a reference silicon wafer. All measurements were performed over a wavelength range of 250 to 1100 nm at 10 nm increment at different angles from 65° to 75° at an interval of 5°. A Cauchy model was used to fit the obtained data. The average value of at least three measurements per sample was reported as the film thickness. The experimental errors were less than 10%.

### 3.8. Zeta Potential and pH

To determine the surface charge of the gold nanoparticles, the zeta potential at the hydrodynamic shear plane [34] was measured using NanoSeries instrument, ATA Scientific. The samples were equilibrated at 25 °C for 2 min and measured three times 100 runs each measurement. The zeta potential value was calculated using Smoluchowski method. To investigate the ability of the various gold containing solutions to protonate the surface coatings, the pH of the solutions was measured using Metrohm pH meter calibrated using standard pH solutions.

### 3.9. ICP-MS

To determine the leaching efficiency of rock samples, the concentration of gold in the leachates was measured using Agilent ICP-MS 7500cs equipped with helium/hydrogen collision cell. The leachates were evaporated overnight to remove HClO to avoid chlorine gas release. The dry samples were reconstituted in 2% nitric acid. The expected final gold concentration of the solution was below 900 ppb (~1200 times dilution). The ICP-MS analysis of rock leachates was performed using the method of standard addition: the rock leachate samples were spiked with a range of concentrations of gold standard (0–500 ppb) to calibrate the concentration of gold in non-spiked sample, thus avoiding any detrimental matrix effect due to the presence of many elements in the leachates. 

### 3.10. LIBS

LIBS was used for the measurement of gold bound to coated silicon wafers. Uncoated and coated wafers incubated with MiliQ water were used as control samples. LIBS was also used for rock leachates and rock powder suspensions. LIBS comprises of a high power (300 mJ) 1064 nm Nd:YAG Q-switched laser configured for a 532 nm output and a Spectrolaser 4000 LIBS analyzer. The system is equipped with a high-precision x-y stepper motor and a spectrometer covering 190 and 950 nm at a resolution of 0.09 nm at 300 nm. All analyses were carried out in ambient indoor conditions using a laser power of 110 mJ per pulse, and a delay of 0.8 ms. Data processing was carried out with the GeoLIBS^TM^ software of the Spectrolaser instrument. An argon purge was used to enhance the gold signal. 

All samples were analyzed in 3 rows of 10 laser pulses, with each row averaged into a single spectrum and separated by 2mm. The 10 laser pulses in each row were evenly spread across 14 mm. The gold peak at 267.6 nm was selected and the peak area was measured between 267.393–267.828 nm.

## 4. Conclusions

We have developed a proof-of-concept for a portable method to detect gold ions and NPs by binding them on functionalized substrates, which were then measured using portable LIBS instrument. The binding of gold ions in a rock leachate on coated substrates has been demonstrated for the first time. In addition, we have demonstrated that hypochlorite efficiently leaches gold from rock powder. For field deployability, PP surface coatings can be mass produced off-site (in labs) and can then be deployed on-site to incubate with gold solution liberated from hypochlorite. After incubation or pre-concentration of gold on substrates for 20 min, LIBS can be utilized on-site for measurement of gold species in solution down to ppb level. 

The analysis of the LIBS gold signal revealed the binding behavior of gold species (ions and NPs) on amine functionalized coatings (PP and WC). The binding of citrate capped gold NPs was slower on all amine coated surfaces compared to gold ions due to the large size of these NPs and low protonation of the amine groups because of the high pH of the NPs solution. By contrast, gold ions in AuCl_3_ solution and gold nugget leachate exhibit faster binding due to small size of the gold ions and effective protonation of the amine groups by the low pH solutions. After 1 h of incubation, gold NPs were still far from saturation even at high concentration of 30 ppm due to its slower binding kinetics whereas gold ions showed saturation at 15 ppm due to faster binding kinetics. Furthermore, a higher amount of gold ions was bound to PP coatings compared to WC coatings due to the higher number of binding sites (amine and imine groups) present in the PP coating relative to the WC coating. 

The binding behavior of the gold species governs the LOD of gold detection with LIBS for pure gold solutions. The slow binding of NPs results in high LOD of 1 ppm whereas the fast binding of gold ions yields low LOD of tens of ppb for both PP and WC coatings. Moreover, for pure gold ion solutions with low gold concentration <1 ppm, both PP and WC coatings resulted in the same gold binding efficiency. By contrast, for rock leachates with <1 ppm gold, PP coating was observed to be more effective than WC coating in binding gold. This is due to the presence of other elements in the complex rock leachates, which compete for binding sites of the surface coatings. The larger number of amine and imine groups of the PP coating provides a larger number of binding sites for metal ions compared with WC coating. Therefore, PP coating is advantageous for gold sensing of complex solutions.

The pre-concentration of gold ions on PP coatings allowed detection of approximately 300–500 ppb of gold in a leachate of rock powder containing 40–50 ppm gold. Incubation of PP coating with rock leachate for 20 min was found to be sufficient for detection of gold in rock leachates. However, without pre-concentration, portable LIBS could not detect gold, even for rock powder suspensions and rock leachates with low dilution factor. The improvement of the leaching process leading to a decrease in the dilution factor as well as the modification of the coating chemistry has the potential to decrease the LOD of gold from rock leachates. Such improvements in pre-concentration and leaching portend a new portable analytical method for the detection of gold in the field, e.g., at a mineral exploration drill rig. 

The reported proof-of-concept method could also be used for the detection of other metal ions using the same portable technique, i.e., LIBS. Furthermore, the method reported has the potential to open new avenues in assessing the nanotoxicity (by detecting different elements) in different environments such as waste, sea, lake and river water. 

## Figures and Tables

**Table 1:** Ratio of functional groups of the PP and WC coatings with-out gold incubation as analyzed from C1s peak. All the XPS data was plotted with a standard error of 5%.

	X	PP	WC
Functional group ratio: X/ΣX	aliphatic carbon [C-H or C-C]	58%	86%
	primary amines [C-NH_2_], secondary amines [C-NH-C], traces of C-O bonds	20%	7%
	imines (C=N), traces of nitrile (C≡N)	18%	0%
	C=O bonds from contamination	5%	7%
Ratio relative to carbon	amine to aliphatic carbon ratio	34%	8%
	imine to aliphatic carbon ratio	32%	0%

**Table 2:** Calculated surface coverage for gold ions and gold NPs in solutions at different gold concentration used for incuba-tion. Coverage of 1 indicates that the substrate surface is completely covered with gold ions or NPs.

Gold Concentration in Solutions Used for Incubation	Calculated Surface Coverage	
	for Gold Ions	for Gold NPs
0.16 ppm	0.1	0.001
1 ppm	0.8	0.005
15 ppm	11.6	0.074
30 ppm	23.3	0.148

**Table 3:** Expected gold concentration in rock leachates (for complete leaching) and rock suspensions according to the gold content provided by rock sample supplier and dilution used. ICP-MS detected gold concentration in rock leachates.

	Rock Leachate			Rock Powder Suspension
	Expected Gold Concentration (ppb)	ICP-MS Detected Gold Concentration (ppb)	Leaching Efficiency (%)	Expected Gold Concentration (ppb)
62e	130	120	92 ± 7	9370
SN74	242	196	81 ± 7	8981
SP72	467	219	47 ± 2	18,160
Oreas 991	391	309	79 ± 4	47,040
SQ88	1032	515	50 ± 2	39,720

## Supplementary Materials

The following are available online at https://www.mdpi.com/1424-8220/20/2/492/s1, Table S1: Elements present in rock leachates, Table S2: LIBS signal on the PP and WC surfaces incubated with rock leachates, Table S3: pH of various gold solutions. Measurement error for the samples was up to ±0.16, except for the range of rock leachates having error of ±0.76, Figure S1: Distribution of gold ions and NPs on the surface, Table S4: Calculated surface coverage for gold ions and gold NPs in solutions at different gold concentration used for incubation. Coverage of 1 indicates that the substrate surface is completely covered with gold ions or NPs, Figure S2: Peak Area measurement of rock powders placed directly on adhesive tape, Figure S3: LIBS peak area measurement of WC coated surfaces incubated with rock powder suspensions, Figure S4: LIBS peak area measurement of PP coated surfaces incubated with rock powder suspensions.
