# Supplementary material for: Field Deployable Method for Gold Detection Using Gold Pre-Concentration on Functionalized Surfaces
Source: Sensors (Basel). 2020 Jan 15;20(2):492. doi: 10.3390/s20020492 (PMC7014198; doi:10.3390/s20020492)
Supplement: Supplementary file 1 [file sensors-20-00492-s001.pdf]

Supporting Information

Rapid detection of gold in low-concentration solutions using gold pre-concentration on functionalized substrates

Agnieszka Zuber [1,2,†], Akash Bachhuka [1,2,3,†,*], Steven Tassios [2,4], Caroline Tiddy [2,5], Krasimir Vasilev [5,6] and Heike Ebendorff-Heidepriem [1,2,3,*]

1 Institute for Photonics and Advanced Sensing, School of Physical Sciences, The University of Adelaide, Adelaide 5005, Australia; agnes.zuber@gmail.com
2 Deep Exploration Technologies Cooperative Research Centre, School of Physical Sciences, The University of Adelaide, Adelaide 5005, Australia; steven.tassios@csiro.au (S.T.); caroline.tiddy@unisa.edu.au (C.T.)
3 ARC Center of Excellence for Nanoscale BioPhotonics, The University of Adelaide, Adelaide 5005, Australia
4 CSIRO, Process Science and Engineering, Gate 1, Normanby Road, Clayton 3169, Australia
5 Future Industries Institute, University of South Australia, Mawson Lakes 5095, Australia; krasimir.vasilev@unisa.edu.au
6 School of Engineering, University of South Australia, Mawson Lakes 5095, Australia
† These authors contribute equally in this paper.
* Correspondence: akash.bachhuka@adelaide.edu.au (A.B.); heike.ebendorff@adelaide.edu.au (H.E.-H.)

Info S1. Specification of the rock standards chosen for the analysis

OREAS

62e: Au 9.13ppm, Ag 9.86ppm, Au 9.37ppm, primary state, matrix meta-andesite, mineralisation low sulphidation epithermal, major elements: S 0.4%, Si 30.71%, As 11.3ppm, Fe 2.95%, Ba 343ppm, Cu 68ppm, Li 50ppm, Ba 30ppm, Ca 3.88%.

991: Au 47.04ppm, Cu 20.66wt%, Ag 48.14ppm, primary, concentrate matrix, porphyry copper gold. Major elements reported by the provider: S 30.77%, As 170ppm, Bi <50ppm, Ca 1wt%, Cd <10ppm, Co 122ppm, Fe 27wt%, Mg 0.5wt%, Mo 490ppm, Ni 32ppm, Pb 123ppm, S 30.77wt%, Sb 80ppm

ROCKLABS

SP72: Origin: Feldspar minerals, basalt and iron pyrites with minor quantities of finely divided gold and silver-containing minerals that have been screened to ensure there is no gold nugget effect. Composition: Au 18.16ppm, Ag 83ppm, other uncertified values: SiO_2 55.88%, Al_2O_3 16.21%, Na_2O

4.81%, K_2O 5.61%, CaO 2.86%, MgO 2.55%, TiO_2 0.7%, MnO 0.06%, P_2O_5 0.2%, Fe_2O_3 3.67%, Fe 3.2%, S 3.5%.

SN74: Origin: Feldspar minerals, basalt and iron pyrites with minor quantities of finely divided gold and silver-containing minerals that have been screened to ensure there is no gold nugget effect. Composition: Au 8.981ppm, Ag 51.5ppm, other uncertified values: SiO_2 55.92%, Al_2O_3 16.03%, Na_2O 4.54%, K_2O 5.45%, CaO 3.14%, MgO 3.04%, TiO_2 0.79%, MnO 0.06%, P_2O_5 0.21%, Fe_2O_3 4.18%, Fe 3.0%, S 3.3%.

SQ88: Origin: Feldspar minerals, basalt and iron pyrites with minor quantities of finely divided gold and silver-containing minerals that have been screened to ensure there is no gold nugget effect. Composition: Au 39.72ppm, Ag 160.8ppm, other uncertified values: SiO_2 47.31%, Al_2O_3 13.64%, Na_2O 3.75%, K_2O 4.37%, CaO 2.96%, MgO 2.67%, TiO_2 0.77%, MnO 0.07%, P_2O_5 0.21%, Fe_2O_3 3.71%, Fe 8.8%, S 10%.

HiSilP3: Origin: A highly siliceous matrix with minor quantities of clay, iron pyrites and finely divided gold-containing minerals that have been screened to ensure there is no gold nugget effect. Composition: Au 12.24ppm, other uncertified values: SiO_2 90.35%, Al_2O_3 2.98%, Na_2O 0.08%, K_2O 0.22%, CaO 0.06%, MgO 0.06%, TiO_2 0.14%, MnO 0.01%, P_2O_5 0.2%, Fe_2O_3 0.75%, Fe 1.8%, S 2.0%.

Table 1. Elements present in rock leachates.

**SN74 PP**	Low Fe, High C, H
**SN74 PP**	High C, H
**SN74 WC**	High Fe, Mg, Al, Na
**SN74 WC**	High Fe, Low Mg, Al
**62e PP**	High C, H
**62e PP**	High C, H
**62e WC**	Very High Fe, Low Mg, Ca, Al
**62e WC**	Very High Fe, Low Mg, Ca,
**991 PP**	High C, H, Na
**991 PP**	High C, H, Na Low Fe
**991 WC**	Low Fe, Mg, Ca, Al
**991 WC**	Low Fe, Mg, Ca, Al
**HiSilP3 PP**	High C, H, Low Fe

**HiSilP3 PP**	High C, H, Low Fe
**HiSilP3 WC**	Low Fe, Mg, Ca, Al
**HiSilP3 WC**	Low Fe, Mg, Ca, Al
**SP72 PP**	High C, H, Low Fe
**SP72 PP**	High C, H, Low Fe
**SP72 WC**	Low Fe, Mg, Ca, Al
**SP72 WC**	High Fe, Na, low Mg, Ca, Al

Table S2. LIBS signal on the PP and WC surfaces incubated with rock leachates.

	Gold concentration (ppb) determined by ICP-MS	LIBS peak area	SD	LIBS signal/1ppb
PP SP72	219	13.07	1.3	0.034
	8219	155.8	12.8	0.018
	30219	381.68	52.7	0.012
PP HiSilP3	355	26.29	2.4	0.082
	355	27.64	2.5	
	355	23.75	2.4	
	30355	594.14	69.8	0.02
PP SN74	196	5.5	3	0.023
	196	3.44	7.3	
PP SQ88	515	30.98	3.42	
	515	33.3	3.4	0.042
	515	38.74	4.6	
PP 991	309	9.7	2.9	0.031
	309	13.36	2.7	
	309	13.14	6.4	
PP 62e	120	1.85	0.9	0.015
	120	-0.14	4.5	
	120	3.52	1.0	
WC HiSilP3	355	28.18	12.1	
	355	16.3	9.0	
WC SN74	196	-2.9	1.8	-
WC SQ88	515	3.44	3.7	0.004
	515	3.2	1.7	
WC 991	309	-0.21	4.0	-
	309	2.05	0.8	
	309	-2.02	0.7	
WC 62e	120	0.94	0.6	
	120	-3.76	4.8	

Table S3. pH of various gold solutions. Measurement error for the samples was up to ±0.16, except for the range of rock leachates having error of ±0.76.

	pH
citrate capped NP in water	7.74
$AuCl_3$ dissolved in water	2.51
Gold nugget leachate	4.84
Rock leachates (other than OREAS991)	4.4
Rock leachate OREAS991	1.04

Model S1. Model of gold binding to surface

Gold species of type *k* (*k* = ions or NPs) bind to the surface in such a way, that each species covers a square area of

$$A_{1,k} = D_{1,k}^2 \ , \qquad \text{(Eq. 1)}$$

where $D_{1,k}$ is the diameter of the species (Supplementary figure 1).

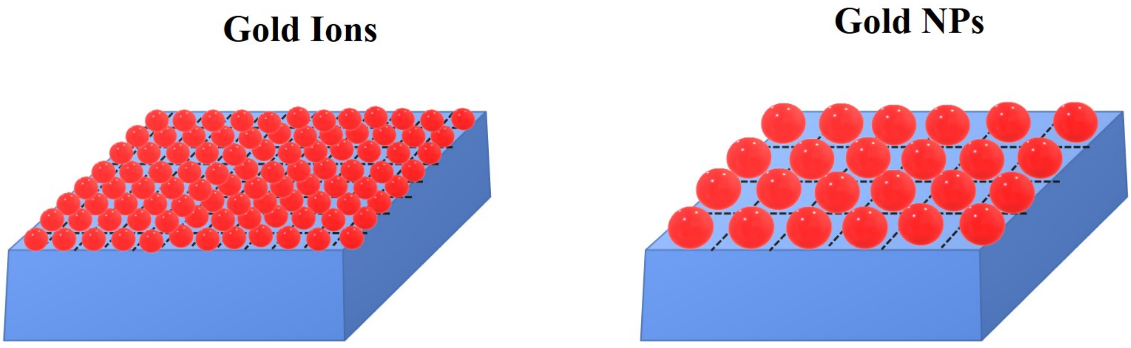

Figure S1. Distribution of gold ions and NPs on the surface.

If there is sufficient free surface available, all gold species in the incubation volume, V_{inc}, are assumed to be bound to the surface, covering an area of

$$A_k = N'_k A_{1,k} = N'_k D_{1,k}^2 \ , \qquad \text{(Eq. 2)}$$

where N'_k is the number of gold species k in the incubation volume. The number of gold species per volume is given by

$$N_k = \frac{N'_k}{V_{inc}} = \frac{\frac{m_k}{m_{1,k}}}{V_{inc}} \ , \qquad \text{(Eq. 3)}$$

where m_k is the mass of gold in V_{inc} and $m_{1,k}$ is the mass if a single gold species. Assuming the density of the gold solutions is 1g/cm^3, m_k is given by the known weight ratio of the solutions, e.g. 1ppm gold is 1µg gold in 1mL incubation volume, i.e. 1ppm gold equals m_k=1µg for our incubation method using 1mL incubation volume. Note that the assumption that all gold species in the incubation volume bind to the surface makes the mass of gold in the incubation volume equal to the mass of gold on the surface area, A_s, provided there is sufficient surface area available. Using N_k, A_k is

$$A_k = N_k V_{inc} A_{1,k} \ . \qquad \text{(Eq. 4)}$$

The surface coverage is defined as

$$C_k = \frac{A_k}{A_s} \ . \qquad \text{(Eq. 5)}$$

where A_s is the substrate area, which is 4cm^2 for our experiments. Using Eqs. 2-4, C_k is calculated by

$$C_k = \frac{m_k}{m_{1,k}} \frac{D_{1,k}^2}{A_s} \ . \qquad \text{(Eq. 6)}$$

Gold ions:

The mass of a single gold ion, $m_{1,ion}$, is calculated from the molar mass of gold, M_{Au}, and the Avogadro number, N_A, by

$$m_{1,ion} = \frac{M_{Au}}{N_A} \quad \text{(Eq. 7)}$$

The diameter of the gold ions on the surface is defined as the distance of gold ions in bulk gold crystal, allowing to use bulk gold density ρ_{Au}, for calculation of gold ion diameter and volume of gold ions on the surface by

$$\rho_{Au} = \frac{m_{Au}}{V_{Au}} = \frac{m_{1,ion}}{V_{1,ion}} , \quad \text{(Eq. 8)}$$

where $V_{1,ion}$ is the volume of a single gold ion, given by

$$V_{1,ion} = \frac{\pi}{6} D_{1,ion}^3 . \quad \text{(Eq. 9)}$$

leading to

$$D_{1,ion} = \left(\frac{6 M_{Au}}{\pi N_A \rho_{Au}} \right)^{\frac{1}{3}} . \quad \text{(Eq. 10)}$$

Using ρ_{Au} = 19.3 g/cm^3, M_{Au} = 196.967g/mol, N_A = 6.022x10^{23}/mol, $D_{1,ion}$ is calculated to be 0.319nm. This value is close to gold atom diameter (van der Waals) of 2*0.166 nm=0.332 nm[1].

Using Eqs. 8 and 9 and k=ion, C_k of Eq. 6 can be rewritten as

$$C_{ion} = \frac{m_{ion}}{\rho_{Au}} \frac{\frac{6}{\pi}}{\frac{D_{1,ion}}{A_s}} . \quad \text{(Eq. 11)}$$

Eq. 11 can be used to calculate the surface coverage from known mass of gold, m_{ion}, in the incubation volume (e.g. 1ppm gold equals m_{ion}=1µg gold in 1mL incubation volume), known gold density, calculated gold ion diameter (Eq. 10) and known substrate area.

Gold NPs:

As the diameter of gold NPs is known, the volume of a single NP, $V_{1,NP}$, can be readily calculated as

$$V_{1,NP} = \frac{\pi}{6} D_{1,NP}^3 \ . \qquad \text{(Eq. 12)}$$

Assuming that the gold NPs have the same density as bulk gold, the mass of a single NP, $m_{1,NP}$, is calculated

$$m_{1,NP} = \rho_{Au} V_{1,NP} = \rho_{Au} \frac{\pi}{6} D_{1,NP}^3 \ . \qquad \text{(Eq. 13)}$$

Using Eqs. 12 and 13, C_k of Eq. 6 is rewritten as

$$C_{NP} = \frac{m_{NP}}{\rho_{Au}} \frac{\frac{6}{\pi}}{\frac{D_{1,NP}}{A_s}} \ . \qquad \text{(Eq. 14)}$$

Similar to gold ions, Eq. 14 can be used to calculate the surface coverage from known mass of gold in the incubation volume (e.g. 1ppm gold equals 1µg gold in 1mL incubation volume), known gold density, known gold NP diameter (Eq. 16) and known substrate area.

Comparison of gold ions and NPs at fixed surface coverage

Using Eqs. 3 and 6, the surface coverage can be expressed in terms of number of gold species on the surface

$$C_k = N_k V_{inc} \frac{D_{1,k}^2}{A_s} \ . \qquad \text{(Eq. 15)}$$

This equation enables to calculate the number ratio of gold NPs to gold ions for the same surface coverage (and the same incubation volume)

for $C_{NP} = C_{ion}$

$$\frac{N_{NP}}{N_{ion}} = \frac{D_{1,ion}^2}{D_{NP}^2} = \left(\frac{50nm}{0.319nm}\right)^2 \sim 25000 \ . \qquad \text{(Eq. 16)}$$

This ratio shows that, for the same surface coverage, the number of gold NPs on the surface is considerably lower than that of gold ions. Specifically, the ratio scales inversely with the square of the corresponding diameter ratio, i.e. the number of gold NPs on surface is ~25,000 times lower compared with gold ions.

The LIBS signal is proportional to the volume of gold on the surface. The volume is proportional to the mass of gold via the gold density

$$V_k = \frac{m_k}{\rho_{Au}} \ . \qquad \text{(Eq. 17)}$$

Equations 11 and 14 express the surface coverage in terms of mass of gold on the surface and the diameter of the corresponding gold species. Using these equations and Eq. 17, the volume ratio of gold NPs to ions bound to the surface for the same coverage can be calculated

for $C_{NP} = C_{ion}$

$$\frac{V_{NP}}{V_{ion}} = \frac{D_{1,NP}}{D_{1,ion}} = \frac{50nm}{0.319nm} = 156.9 \ . \qquad \text{(Eq. 18)}$$

This ratio shows that the volume ratio and thus LIBS signal scales with the diameter ratio. Considering that the volume of gold on the surface equals the number of gold species on the surface multiplied with the volume of a single species,

$$V_k = N_k V_{1,k} \ , \qquad \text{(Eq. 19)}$$

the volume ratio scaling factor is the result of the number of species on the surface scaling with the inverse of the square of diameter ratio, whereas the volume ratio of the single gold species scales directly with the cubic of the diameter ratio.

Surface coverage as a function of gold content in incubation solution

Using Eqs. 11 and 14, and known gold weight ratio of the incubation solutions (which equals m_{ion} or m_{NP} in incubation volume), the surface coverage for different gold concentration of the incubation solutions can be calculated.

Table S4. Calculated surface coverage for gold ions and gold NPs in solutions at different gold concentration used for incubation. Coverage of 1 indicates that the substrate surface is completely covered with gold ions or NPs.

gold concentration in solutions used for incubation (gold mass in 1mL incubation volume)	calculated surface coverage	
	for gold ions	for gold NPs
0.16 ppm (0.16μg)	0.1	0.001
1 ppm (1μg)	0.8	0.005
15 ppm (15μg)	11.6	0.074
30 ppm (30μg)	23.3	0.148

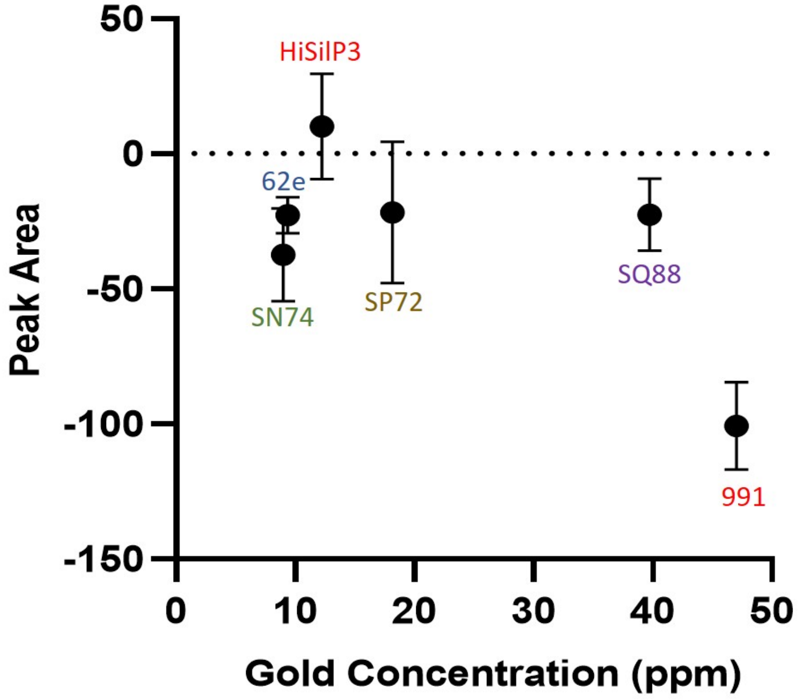

Figure S2. LIBS peak area measurement of rock powders placed directly on adhesive tape.

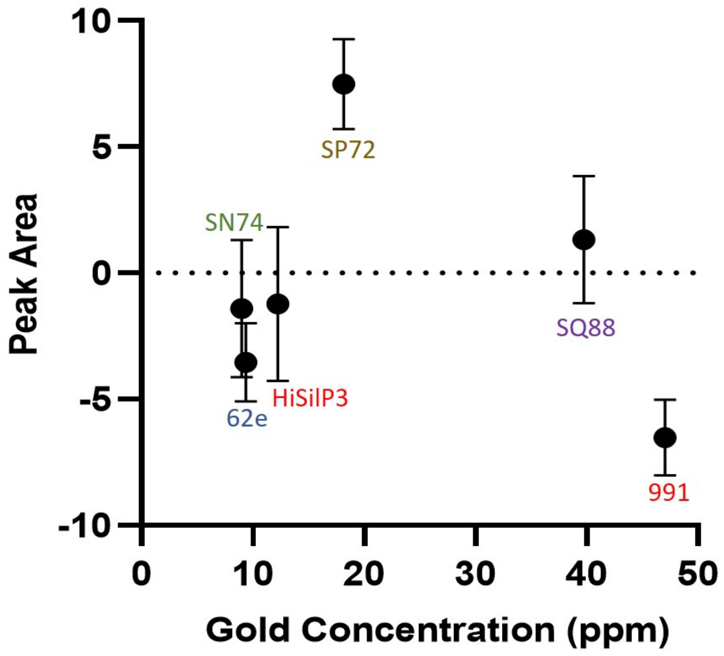

Figure S3. LIBS peak area measurement of WC coated surfaces incubated with rock powder suspensions.

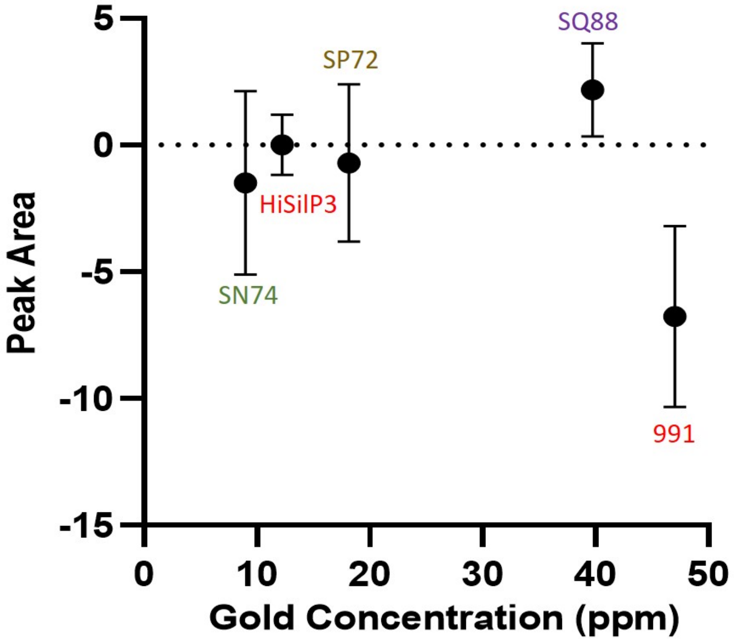

Figure S4. LIBS peak area measurement of PP coated surfaces incubated with rock powder suspensions.

References

1. Bondi A. van der Waals Volumes and Radii. *The Journal of Physical Chemistry*. 1964/03/01 1964;68(3):441-451.
